# Medications for early treatment of COVID‐19 in Australia

**DOI:** 10.5694/mja2.51750

**Published:** 2022-10-23

**Authors:** Adam Polkinghorne, James M Branley

**Affiliations:** ^1^ Pathology West Nepean Hospital Sydney NSW; ^2^ Nepean Clinical School University of Sydney Sydney NSW

## Abstract

- Early treatment of severe acute respiratory syndrome coronavirus 2 (SARS‐CoV‐2) infections can prevent hospitalisation and death in patients with coronavirus disease 2019 (COVID‐19) who have one or more risk factors for serious COVID‐19 progression. While early treatment presents a range of logistical challenges, clinicians are nevertheless aided by a growing number of approved medications for early treatment of COVID‐19.
- Medications include drugs that inhibit SARS‐CoV‐2 viral replication, anti‐SARS‐CoV‐2 monoclonal antibody formulations that provide passive immunisation, and immunomodulatory drugs that suppress the body’s inflammatory response.
- Several drugs with different modes of action are approved in Australia for early treatment of COVID‐19, including nirmatrelvir plus ritonavir, molnupiravir, and monoclonal antibody formulations. Although these drugs are recommended, clinicians are encouraged to remain up to date on current indications, contraindications and the clinical efficacy of these drugs against SARS‐CoV‐2 variants currently circulating in communities.
- Other treatments, including hydroxychloroquine, ivermectin and dietary supplements, have been popularised but are not recommended for early treatment of COVID‐19.
- As new drugs and new data on use of existing approved drugs become available, clinicians face a growing challenge in determining the optimal treatments from the array of options. As it stands, early treatment of COVID‐19 needs to be individualised depending on age, pregnancy status, existing medications, and renal and liver disease status. Future treatments in development might have roles in patients with lower risk profiles and in reducing transmission as we learn to live with SARS‐CoV‐2.

The emergence of severe acute respiratory syndrome coronavirus 2 (SARS‐CoV‐2), the causative agent of coronavirus disease 2019 (COVID‐19), has resulted in a global pandemic with over 500 million confirmed cases and more than 6 million deaths as of April 2022.^1^ Incredible progress has nevertheless been made over the past 2 years in diagnosis, treatment and supportive care for patients with COVID‐19. Vaccination has had a major impact on the severity and clinical outcomes of SARS‐CoV‐2 infection.^2^, ^3^ In Australia, as of May 2022, more than 95% of people over the age of 16 years were fully vaccinated (two doses) and about 69% of the eligible population had received at least one booster dose.^4^ Despite this progress, unvaccinated populations still pose considerable challenges to clinicians in Australia and elsewhere around the world. Certain populations within the community are of ongoing concern, given that serious COVID‐19 has been predominantly experienced among groups with risk factors such as: older age (>60 years); obesity; cardiovascular, respiratory, haematological, oncological and renal disease; pregnancy; and conditions that cause patients to be immunocompromised.^5^ In addition, marginalised groups with poor access to health care (eg, Indigenous people, immigrants, people with mental health conditions, homeless people, and people who misuse substances) are at particular risk of severe COVID‐19 outcomes.

COVID‐19 consists of two parts: an initial phase in which patients have an asymptomatic infection or symptoms consistent with a mild to moderate viral upper respiratory disease and, potentially, a later phase of severe respiratory illness in which patients may develop hypoxaemia that can progress to hypoxic respiratory failure.^6^ Where progression occurs, it is characterised by systemic hyperinflammation driven by release of proinflammatory cytokines,^6^ and results in substantial mortality and morbidity. Initial COVID‐19 therapies were focused on modifying the immune response during the later stages of severe disease, in part due to a lack of treatments that acted specifically against SARS‐CoV‐2 infection.^7^ Immunomodulatory drugs that have been used to treat severe COVID‐19 include drugs that target interleukin‐6 (eg, tocilizumab) and Janus kinases involved in cytokine‐mediated signalling (eg, baricitinib), and other immunosuppressive drugs such as corticosteroids (eg, dexamethasone).^7^ By their nature, these treatments are most useful for severely unwell hospitalised patients, but they have notable risks of adverse effects. Although often lifesaving, these late treatments would not be necessary if early suppression of viral replication prevented the development of a cytokine cascade. Fortunately, as the pandemic has progressed, a range of specific and non‐specific COVID‐19 treatments have emerged. These treatments appear to have efficacy in two groups of patients: those presenting to hospital symptomatically but not yet requiring oxygen support, and those in the community who are in at‐risk groups.

In this article, we review the use of early treatment options approved in Australia to treat patients with mild to moderate COVID‐19 and discuss areas where more research is needed. We also discuss the practical challenges of early treatment of COVID‐19.

## Drugs for early treatment of COVID‐19

Treatment options suitable for administration to individuals with mild to moderate COVID‐19 can be divided into three categories: antivirals that non‐specifically act on or specifically target SARS‐CoV‐2; monoclonal antibodies (mABs) used to provide passive immunotherapy; and immunomodulatory drugs such as inhaled corticosteroids. In the following section, we summarise the findings of a literature review that we conducted on a selection of drugs used for early treatment of adult patients with COVID‐19, highlighting mode of action, evidence of clinical efficacy, indications and common contraindications. We selected these drugs based on extensive clinical experience in treating patients during the COVID‐19 pandemic in Western Sydney between 2020 and 2022 and from those approved for administration to patients with COVID‐19 in Australia. Relevant peer‐reviewed original research, reviews and meta‐analyses published between January 2020 and May 2022 were identified by searching the MEDLINE database.

### Nirmatrelvir plus ritonavir

Nirmatrelvir plus ritonavir is an oral combination antiviral drug. Nirmatrelvir is a second‐generation protease inhibitor with strong antiviral SARS‐CoV‐2 reactivity, and ritonavir is a protease inhibitor that strongly suppresses host liver enzymes (cytochrome P450 3A4) that can metabolise nirmatrelvir.^8^ Ritonavir thereby assists in achieving peak nirmatrelvir concentrations and an extended half‐life for nirmatrelvir in the body.

In vitro studies have shown that nirmatrelvir potently inhibits SARS‐CoV‐2 replication,^9^ and has efficacy against the major SARS‐CoV‐2 variants identified to date (Beta, Delta and Omicron).^10^, ^11^ A living systematic review of published clinical trials investigating nirmatrelvir plus ritonavir administration for COVID‐19 has concluded that nirmatrelvir plus ritonavir reduces the risk of hospital admissions in patients with non‐severe disease by 80% with a moderate level of certainty (7 in 1000 cases; 95% CI, 2–17) compared with standard care (43 in 1000 cases).^12^ However, recent reports have highlighted the potential for rapid relapse of symptomatic SARS‐CoV‐2 infection following initial treatment with nirmatrelvir plus ritonavir,^13^ suggesting that prolongation of treatment might be required to prevent this phenomenon in certain risk groups.

In Australia, nirmatrelvir plus ritonavir is recommended for administration within 5 days of symptom onset in SARS‐CoV‐2‐positive adults with risk factors for disease progression.^14^ Concern exists over potentially dangerous interactions between ritonavir and a diverse range of drugs, including antiarrhythmics, anticoagulants and many other medications.^15^ Some of these reactions are potentially serious or even life‐threatening, whereas many are relatively trivial. As many people in the target population have cardiovascular risk factors, changes in the use of drugs that are used to manage these conditions might be needed during ritonavir therapy (eg, treatment interruption, dose reduction or monitoring).

### Remdesivir

Remdesivir is a broad‐spectrum antiviral drug with demonstrated activity against a diversity of coronaviruses,^16^ including SARS‐CoV‐2.^17^ It is an adenosine analogue that targets the SARS‐CoV‐2 RNA‐directed RNA polymerase required for transcription and translation of the viral genome.^18^ A systematic review and meta‐analysis found that remdesivir use is probably associated with a significant reduction in the risk of hospital admission in patients with non‐severe disease of about 70% (14 in 1000 cases; 95% CI, 3–37) compared with standard care (43 in 1000 cases).^12^ A separate analysis also found that remdesivir use reduced the relative risk of mortality in patients requiring supplemental oxygen (0.77; 95% CI, 0.50–1.19) and those requiring mechanical ventilation (0.89; 95% CI, 0.79–0.99) compared with standard care or placebo.^19^

In Australia, intravenous remdesivir was provisionally approved for COVID‐19 treatment in adults and children aged ≥ 12 years who require supplemental oxygen.^20^ Based on the results of a randomised controlled trial,^21^ this indication was expanded to include adults in the community with confirmed COVID‐19 who are at risk of hospitalisation and are within 7 days of symptom onset. However, since remdesivir requires intravenous administration, its use in general practice is limited compared with approved oral treatments.

Remdesivir appears to be well tolerated in most patients, with occasional observations of bradycardia. Other adverse effects — such skin rashes, elevated liver enzyme levels, anaemia and acute kidney injury — are uncommon.^22^ Because of the limitations of other COVID‐19 drugs for use in patients with end‐stage renal disease and pregnant women, judicious use of remdesivir may be of value in these populations.

### Molnupiravir

Molnupiravir is a ribonucleoside analogue that targets the SARS‐CoV‐2 RNA‐directed RNA polymerase, thereby increasing the frequency of viral RNA mutations and the production of faulty viral particles, and can be given orally.^23^ In rodents, molnupiravir therapy has been shown to significantly reduce replication and pathogenesis of SARS‐CoV‐2,^24^, ^25^, ^26^ to the extent that transmission to naïve animals is blocked.^25^ Systematic review and meta‐analysis of the results of clinical trials of molnupiravir has shown that treatment probably leads to a reduction in the risk of hospital admission in patients with non‐severe disease of about 50% (24 in 1000 cases; 95% CI, 14–38) compared with standard care (43 in 1000 cases).^12^ The same report also noted that molnupiravir treatment appears to improve the time to symptom resolution (6.6 days; 95% CI, 5.1–8.3) compared with the time to symptom resolution following standard care (9.9 days).^12^

Molnupiravir is approved in Australia for the treatment of adults with COVID‐19 who are at increased risk of hospitalisation or death.^27^ Molnupiravir is contraindicated in pregnant women due to concerns that this drug may cause fetal harm. This recommendation is based on an overdosing study that investigated oral administration of molnupiravir and found that it induced embryofetal lethality and teratogenicity in pregnant rats.^27^ As a precautionary principle, patients receiving molnupiravir treatment are advised to use contraceptives during treatment and for 3 months for men and 4 days for women after the last dose.

### Inhaled corticosteroids

Inhaled corticosteroids such as budesonide and ciclesonide are anti‐inflammatory drugs that have been historically used for treatment of breathing disorders, such as chronic obstructive pulmonary disease^28^ and asthma,^29^ and other disorders.

A meta‐analysis of the results of open‐label clinical trials has shown a high probability that inhaled corticosteroids significantly reduce the risk of hospitalisation compared with controls (relative risk, 0.44; 95% CI, 0.12–1.70).^30^ This observed risk reduction was more modest when the analysis was restricted to placebo‐controlled studies (relative risk, 0.90; 95% CI, 0.22–3.71). Interestingly, suppression of inflammation might not be the only mechanism by which inhaled corticosteroids act against SARS‐CoV‐2. In vitro studies have shown that budesonide^31^ and ciclesonide^32^ inhibit viral RNA replication and transcription, with activity against a range of coronaviruses, suggesting that the beneficial effects of inhaled corticosteroids in the treatment of COVID‐19 could be the result of both an immunosuppressive effect and a direct antiviral effect. In Australia, inhaled corticosteroids (budesonide and ciclesonide) are conditionally recommended for treatment of patients with COVID‐19 who have one or more risk factors for disease progression and are within 14 days of symptom onset.^14^

### Monoclonal antibodies with specificity for SARS‐CoV‐2

Passive immunotherapy has emerged as a successful treatment for mild and moderate cases of COVID‐19.^33^ The mABs used target either the N‐terminal domain of the SARS‐CoV‐2 spike glycoprotein or the viral receptor‐binding domain. Several mAB preparations have been approved for treatment of patients with early (within 5–7 days of symptom onset) mild to moderate COVID‐19 in Australia, including sotrovimab and casirivimab plus imdevimab.

A systematic review and meta‐analysis of the efficacy of SARS‐CoV‐2‐specific mAB therapy in patients with COVID‐19 found that both sotrovimab treatment (odds ratio, 0.20; 95% CI, 0.08–0.48) and casirivimab plus imdevimab treatment (odds ratio, 0.29; 95% CI, 0.20–0.42) were significantly associated with a reduced risk of hospitalisation compared with standard care.^34^ Highlighting the further clinical potential for mAB therapy, tixagevimab plus cilgavimab has been shown to be effective when used as pre‐exposure prophylaxis, with a recent study showing that a single intramuscular dose provided patients with a 76.7% (95% CI, 46.0%‐90.0%) relative risk reduction of symptomatic SARS‐CoV‐2 infection compared with placebo.^35^ This latter mAB formulation has been subsequently approved in Australia for pre‐exposure prophylaxis of COVID‐19 in adults.^36^

The emergence of the Omicron strain and other SARS‐CoV‐2 variants has, unfortunately, placed limits on the clinical efficacy of many of the current mABs for COVID‐19. The realisation of this phenomenon began when researchers found that both neutralising mABs (targeting the SARS‐CoV‐2 receptor‐binding and N‐terminal domains) and serum‐derived polyclonal antibodies (from convalescent patients and COVID‐19 vaccine recipients) had reduced activity against genetically distinct SARS‐CoV‐2 variants.^37^ Subsequent studies specifically targeting the Omicron SARS‐CoV‐2 variant have demonstrated that, in vitro, this variant has reduced binding affinity to many of the therapeutic mABs approved for COVID‐19 treatment.^38^, ^39^ Alarmingly, a recent Australian study showed that mutations in SARS‐CoV‐2 spike proteins, linked to in vitro resistance to commercially available mABs, could also develop in vivo due to administration of neutralising mABs.^40^ High‐throughput B cell screening approaches hold some opportunity to compete in this “pathogen arms race”, with at least one new mAB formulation (bebtelovimab) identified that retains neutralising activity against all known SARS‐CoV‐2 variants to date.^41^ Alternatively, existing specific mABs may have the potential to regain efficacy as SARS‐CoV‐2 evolves to meet the deployment of newer mAB treatments when the selective pressure of older mAB treatments is removed.

The variable susceptibility of SARS‐CoV‐2 variants to these therapeutic mABs nevertheless poses substantial challenges to clinicians devising effective early treatment options for patients with COVID‐19. Most clinical units do not have access to SARS‐CoV‐2 genotyping tools in a clinically relevant timeframe. As a result, decisions on which therapeutic mABs to administer to patients are anticipated to be made based on a combination of up‐to‐date knowledge on the molecular epidemiology of SARS‐CoV‐2 variants circulating in each community and, in the absence of experimental data, in silico predictions of mAB efficacy based on careful study of the viral epitopes targeted by the various mABs.^42^ Given the reduced binding affinity reported for certain mABs, clinicians could opt for other treatment options if available. If other options are not available, an increase in the dosage might compensate for reduced binding affinity.

### Drugs not supported for clinical use in the early treatment of COVID‐19

The absence of effective drugs for SARS‐CoV‐2 infection at the start of the pandemic has led to considerable interest in the potential to repurpose existing drugs and supplements to treat patients with COVID‐19. To date, clinical trials do not exist or have failed to show any improvement in survival or hospitalisation for patients with COVID‐19 for approved drugs such as ivermectin,^43^ hydroxychloroquine^44^, ^45^ and azithromycin^46^ and for dietary supplements such as vitamin C,^47^ vitamin D^48^ and zinc.^49^ Outside of research studies, these treatments are not recommended for use in the treatment of patients with COVID‐19 in Australia.^14^

### Goals for developing new drugs for early treatment of COVID‐19

The current array of approved drugs for early treatment of COVID‐19 has dramatically improved the treatment options available, particularly when compared with the start of the pandemic. In addition, in August 2022 there were more than 100 active registered clinical trials evaluating new and existing therapies for the treatment of COVID‐19 globally (based on a search for COVID‐19 trials on https://clinicaltrials.gov). At this stage of the pandemic, it is nevertheless worth considering the clinical gaps that still exist in the repertoire of treatment options available for early treatment of COVID‐19 (Box 1).

Box 1 **Targets for further COVID‐19 treatment research**

**Clinical gaps and target groups for new COVID‐19 drugs**

- New drugs with indications for patients with COVID‐19 who have liver or renal disease
- New drugs with indications for breastfeeding and pregnant women
- Drugs that minimise “pill burden” of COVID‐19 medications

**Areas where more data are needed for approved COVID‐19 drugs**

- Real‐world clinical studies in largely vaccinated populations, including potential markers of clinical efficacy beyond death and hospitalisation
- Combination therapy studies
- Cost–benefit analyses of COVID‐19 drug prescription in vaccinated populations
- Clinical efficacy in pregnant women, children and immunosuppressed patients
- Benefits of COVID‐19 drugs in reducing the incidence of long COVID

COVID‐19 = coronavirus disease 2019.

In Australia, the overwhelming number of deaths that continue to be reported are in Australians aged 70 years and over.^50^ Most have multiple comorbidities, placing these individuals at a higher risk of disease progression while also challenging efforts to identify suitable treatments compatible with their complex polypharmacy requirements. Problems in prescribing treatments for older patients are further exacerbated by the substantial “pill burden” associated with many of the current oral COVID‐19 drugs. Simple one‐pill‐per‐day treatments would thus greatly enhance the feasibility of and adherence to COVID‐19 treatments offered in the community to older people and people in other risk groups. Our review of the drugs available for early treatment of COVID‐19 has also highlighted that many of these approved drugs are contraindicated in patients medicated for existing comorbidities such as renal and liver failure. Many of the same drugs are also not recommended for treatment of pregnant or breastfeeding women.

To date, hospitalisations (as a proxy for disease progression) and death rates continue to be the primary outcomes measured in terms of the efficacy of early treatments for COVID‐19. While this was of particular importance earlier in the pandemic, in part to ensure that our health systems were not overwhelmed by COVID‐19 cases, the fact that patients in Australia continue to be admitted to hospital for symptomatic COVID‐19 seems to justify their use as key parameters in assessing the efficacy of new drug treatments. But what about other parameters? From a health services perspective, length of hospital stay is and will become an increasingly important parameter as we become more accustomed to “living with COVID‐19”. In animal models, the ability to reduce transmission between infected and uninfected housemates has been used as an efficacy parameter in studies of COVID‐19 treatments.^10^

In addition to the potential for new treatments, there is still much to be learned about the clinical application and potential use of drugs currently approved for COVID‐19 treatment (Box 1). For instance, most clinical trials of COVID‐19 drugs have been conducted in adults but have excluded patients in many important risk groups. This has resulted in a general lack of safety data available on the use of COVID‐19 drugs during pregnancy and breastfeeding.^51^ There is also a general lack of large‐scale data on the best options for early treatment of COVID‐19 in children, particularly those with important underlying medical problems, and immunosuppressed patients. The intersection between approved COVID‐19 drug treatments and “long COVID‐19” syndrome^52^ is also largely unclear. Health economists around the world are also beginning to ask important questions about the cost‐effectiveness of certain approved COVID‐19 treatments. While not uniform in their agreement, studies to date have largely suggested that treatment of patients with COVID‐19 who are at risk of disease progression is cost‐effective,^53^, ^54^, ^55^ but the benefit–cost ratio might decline in high‐income settings as the risk of COVID‐19 progression decreases throughout communities. An expansion of these studies is needed particularly since they have largely relied on the results of clinical efficacy trials, so they have not used real‐world data on the clinical benefits of approved COVID‐19 drugs in the general, largely vaccinated population.

### A basic early COVID‐19 treatment algorithm

In Australia, health authorities attempt to provide up‐to‐date clinical guidelines and flowcharts to help clinicians select the “correct” treatment options for current SARS‐CoV‐2 variants in the community.^14^ Nevertheless, questions remain regarding which drugs to choose when there are often several correct treatment options available. For example, under current guidelines, some patients can simultaneously qualify for nirmatrelvir plus ritonavir (with medication modification), molnupiravir, and an appropriate mAB (the latter depending on the variants circulating in the population). The challenge for the clinician is to skilfully select the best option for the individual patient. From a virological perspective, combination therapy would be an attractive option to maximise individual therapy and prevent resistance, particularly for patients who are likely to struggle with viral clearance, such as severely immunosuppressed patients and those with other haematological disorders. Unfortunately, there is a general lack of studies evaluating the efficacy of combination treatments and, in their absence, clinicians will need to rely on their own clinical experience and that of their colleagues for the foreseeable future.

A simple COVID‐19 treatment algorithm is shown in Box 2. This algorithm is based on recommendations from the New South Wales Government Agency for Clinical Innovation as of 12 August 2022.^56^ Specific recommendations vary between Australian states and may change over time, so clinicians should refer to up‐to‐date approved guidelines for their own jurisdiction for early treatment of COVID‐19.^14^, ^57^, ^58^, ^59^ Patients presenting with mild or moderate upper respiratory tract symptoms should be tested by polymerase chain reaction and/or a rapid antigen test to confirm SARS‐CoV‐2 positivity. A risk assessment for early treatment of COVID‐19 should consider a variety of patient factors, including current COVID‐19 vaccination status, age, presence of immunosuppression, and/or presence of other known risk factors for COVID‐19 progression (Box 2). It is recommended that treatment with most approved early COVID‐19 drugs begins within 5 days of symptom onset or COVID‐19 diagnosis, and that treatment with mABs and remdesivir begins within 7 days. As has already been discussed, the use and choice of mABs should be informed by knowledge regarding SARS‐CoV‐2 variants circulating in the community. Although certain COVID‐19 drug indications and contraindications are described in Box 2, we recommend referring to approved online resources for specific patient groups — these provide indications, contraindications and potential adverse effects of approved COVID‐19 medications and are updated in real time.^14^, ^57^, ^58^, ^59^ In certain risk groups, prolongation of therapy is also recommended to reduce the risk of relapse of symptomatic COVID‐19.

Box 2 A simple algorithm for early treatment of COVID‐19, based on guidance from the New South Wales Government Agency for Clinical Innovation^56^ ^,^*

**Figure:**
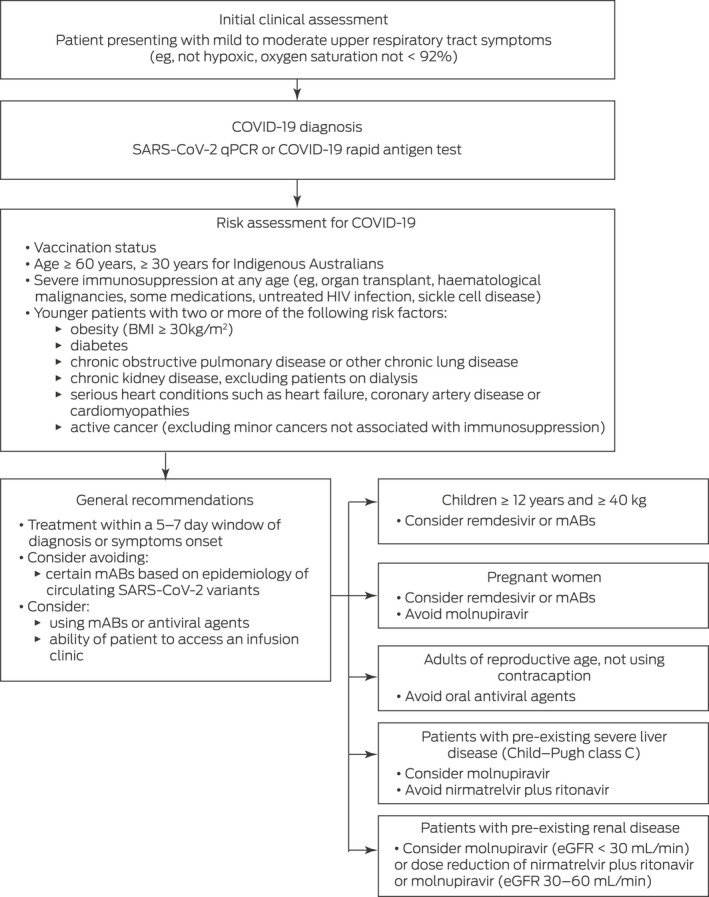
BMI = body mass index. COVID‐19 = coronavirus disease 2019. eGFR = estimated glomerular filtration rate. mABs = monoclonal antibodies. qPCR = quantitative polymerase chain reaction. SARS‐CoV‐2 = severe acute respiratory syndrome coronavirus 2. * As of 12 August 2022. Clinicians are strongly advised to refer to up‐to‐date local approved guidelines for specific recommendations.

## Conclusion

Our review has focused on early treatment options for COVID‐19, because early interventions hold the best opportunity to prevent more serious outcomes in patients who are at risk of disease progression. Unfortunately, early treatment also poses substantial logistical and practical challenges. First, for many COVID‐19 drugs, it is recommended that treatment begins in the first 5 days of illness in patients with risks. Early identification of these patients, selection of appropriate therapy, route of administration, location of care and escalation to hospital care, if required, all require considerable levels of effective cooperation and communication between local clinicians and COVID‐19 management centres, where present. Safe administration of these early treatments also requires that clinicians be well informed of the potential drug interactions associated with the use of SARS‐CoV‐2 antivirals. Quick and user‐friendly web applications such as the COVID‐19 drug interactions resource^59^ and related sites are thus critical in informing decisions in a variety of health settings where clinicians will see patients with early COVID‐19.

Our review is limited by the rapid evolution of COVID‐19 treatment options. In two and a half years we have progressed from no vaccine and no effective treatments for COVID‐19 to what is sometimes a confusing array of options for a largely vaccinated population. In addition, the number of available COVID‐19 treatment options will continue to grow over the coming months as new treatments that are currently being trialled become available. While hopefully supporting the treatment of COVID‐19 among a broader range of patient groups, an increase in the overall number of different options available would also appear to be our best chance at being able to live alongside the rapidly evolving SARS‐CoV‐2.

**Authors’ note:** The results of a recently concluded large randomised controlled trial, currently available as a preprint (https://papers.ssrn.com/sol3/papers.cfm?abstract_id=4237902 [viewed 14 October 2022]), have cast doubts on the efficacy of molnupiravir in reducing hospitalisation and death in patients with COVID‐19. In both molnupiravir treatment and control groups, the observed rate of hospitalisation and death was only 0.8%. Patients treated with molnupiravir had a measurably improved time to first recovery, consistent with other reports.^12^

[Correction added on 26 October 2022 after first online publication: reference 43 has been updated to replace a retracted article with an alternative citation. The change does not affect the content of this narrative review.]

## Open access

Open access publishing facilitated by The University of Sydney, as part of the Wiley – The University of Sydney agreement via the Council of Australian University Librarians.

## Competing interests

No relevant disclosures.

## Provenance

Commissioned; externally peer reviewed.
